# VEGF and Pleiotrophin Modulate the Immune Profile of Breast Cancer

**DOI:** 10.3390/cancers2020970

**Published:** 2010-05-26

**Authors:** Kristi D. Lynn, Christina L. Roland, Rolf A. Brekken

**Affiliations:** 1 Division of Surgical Oncology, Department of Surgery; Hamon Center for Therapeutic Oncology Research, University of Texas Southwestern Medical Center, Dallas, TX, 75390-8593, USA; E-Mails: kristi.lynn@utsouthwestern.edu (K.D.L.); CRolan@parknet.pmh.org (C.L.R.); 2 Department of Pharmacology, University of Texas Southwestern Medical Center, Dallas, TX, 75390-8593, USA

**Keywords:** VEGF, pleiotrophin, macrophage, anti-VEGF, angiogenesis

## Abstract

Angiogenesis, the sprouting of the existing vascular network to form new vessels, is required for the growth of solid tumors. For this reason, the primary stimulant of angiogenesis, vascular endothelial growth factor-A (VEGF), is an attractive target for tumor therapy. In fact, there are currently numerous anti-VEGF therapies in clinical development for the treatment of various cancers, including breast cancer. VEGF signals through two primary VEGF receptors, VEGFR1 and VEGFR2. VEGFR2 is the primary angiogenic receptor, and VEGFR1 has been implicated in macrophage chemotaxis and tumor cell survival and invasion. It has only been appreciated recently that the VEGFRs are expressed not only on endothelial cells and tumor cells but also on many host immune cells. Therefore, to better understand the effects of anti-VEGF therapy it is important to consider the effects of VEGF on all cells in the tumor microenvironment, including immune cells. Bevacizumab (Avastin^®^, Genetech), which binds VEGF and inhibits interaction with VEGFR1 and VEGFR2, was approved for the treatment of metastatic HER2/NEU-negative breast cancer in 2008, however, the majority of human mammary tumors are either innately resistant or will acquire resistance to anti-VEGF therapy. This suggests that these tumors activate alternate angiogenesis pathways. Pleiotrophin (PTN) is an important angiogenic cytokine in breast cancer and is expressed at high levels in approximately 60% of human breast tumors. PTN functions as an angiogenic factor and promotes remodeling of the tumor microenvironment as well as epithelial-mesenchymal transition (EMT). In addition, PTN can have profound effects on macrophage phenotype. The present review focuses on the functions of VEGF and PTN on immune cell infiltration and function in breast cancer. Furthermore, we will discuss how anti-VEGF therapy modulates the immune cell profile.

## 1. Introduction

Breast cancer is the most frequently diagnosed malignancy in women in North America. Advancements in standard treatment regimens have improved the overall outlook for breast cancer patients in recent years; however, 40,000 women a year succumb to this disease [1]. Breast cancer initiates when mammary epithelial cells acquire mutations in genes that regulate cell proliferation, survival, polarity, and differentiation (reviewed in [2]). However, a growing body of evidence indicates that the stromal cell response to these malignant cells is required for the tumor to advance past the hyperplastic stage (reviewed in [2,3]). Cells of the tumor stroma include host fibroblasts, endothelial cells, and immune cells, all of which are known to promote carcinogenesis.

Angiogenesis, the sprouting of an existing vascular network to form new vessels, is required for the growth of solid tumors [4]. For this reason, tumor angiogenesis is an attractive target for tumor therapy. Many of the current anti-angiogenic therapies target vascular endothelial growth factor-A (VEGF), a primary stimulant for angiogenesis [5]. VEGF binds to and activates two primary VEGF receptors, VEGFR1 and VEGFR2. VEGFR2 is the primary angiogenic receptor, while the function of VEGFR1 is less defined (reviewed in [6]). VEGFR1, however, has been implicated in macrophage chemotaxis [7], tumor cell survival [8] and invasion [9]. It is important to note that the VEGFRs are expressed on endothelial cells, tumor cells and on many host immune cells. Therefore, to better understand the effects of anti-VEGF therapy it is important to consider the effects of VEGF on all cells in the tumor microenvironment.

The majority of human mammary tumors are either innately resistant or will acquire resistance to anti-VEGF therapy [10], suggesting that these tumors activate alternate angiogenesis pathways. The alternate angiogenic factors fibroblast growth factor (FGF 1 and 2), ephrin A1 and 2, angiopoietin (Ang-1 and 2), placental growth factor (PlGF), stromal cell-derived factor 1 alpha (SDF-1α), and others, have been implicated in resistance to anti-VEGF therapy (reviewed in [11]). Furthermore, many of these affect immune cell infiltration and function [12,13,14,15,16,17,18,19,20,21,22,23,24]. In this review we focus on two angiogenic cytokines: VEGF and pleiotrophin. Pleiotrophin (PTN) is a less studied but important angiogenic cytokine in the mammary tumor microenvironment (reviewed in [25]). Interestingly, anti-VEGF therapy can modulate PTN expression in multiple pre-clinical mouse models [26]. PTN, an 18 kDa heparin-binding cytokine, is expressed extensively during development, but expression in adult tissues is restricted except during times of inflammation and remodeling [27,28,29,30]. The primary receptor for PTN is receptor protein tyrosine phosphatase (RPTP)β/ζ. RPTPβ/ζ maintains steady-state phosphorylation levels of a number of substrates including anaplastic lymphoma kinase (ALK), β-catenin, β-adducin and others under normal cellular conditions. When bound by PTN, RPTPβ/ζ is inactive and can no longer keep substrate phosphorylation in check. The result is a net increase in substrate phosphorylation following PTN stimulation, which can promote cell proliferation, migration, differentiation, and transformation [31,32,33,34,35].

PTN is an important angiogenic cytokine in many models of breast cancer [25] and is expressed highly by approximately 60% of primary breast tumors [32,36]. When MCF-7 human breast cancer cells were engineered to express high levels of PTN, tumor progression and angiogenesis was enhanced [37]. Additionally, expression of a dominant negative PTN by MDA-MB-231 human breast cancer cells abrogated angiogenesis and progression to malignancy [38]. Finally, when PTN was over-expressed in the mammary fat pad of MMTV-PyMT transgenic mice, a well characterized transgenic breast cancer model, angiogenesis and tumor progression was accelerated significantly [39]. PTN functions as an angiogenic factor and promotes remodeling of the tumor microenvironment and epithelial-mesenchymal transition (EMT) [35,39]. These studies suggest that PTN is an attractive therapeutic target in breast cancer; however, more information is needed concerning the effects of PTN on the tumor microenvironment including recruitment and activation of immune cells. 

The present review focuses on the functions of the angiogenic factors VEGF and PTN on immune cell infiltration and function in breast cancer. Furthermore, we will discuss how anti-VEGF therapy modulates the immune cell profile (Table 1).

## 2. Bone Marrow

Bone marrow consists of a diverse population of cells including hematopoietic stem cells (HSCs), endothelial cells (ECs), chondroblasts, osteoblasts, and other stromal cells. VEGF is expressed by a number of cells in bone marrow, including HSCs, and serves a variety of functions [40,41]. Interestingly, VEGF-deficient HSCs and bone marrow mononuclear cells are unable to form colonies *in vitro* or repopulate lethally irradiated hosts, suggesting that VEGF regulates HSC survival [41]. Further studies indicate that VEGF maintains HSC pluripotency and regulates survival through an internal autocrine loop [40]. Receptor tyrosine kinase inhibitors, such as sunitinib (Sutent^®^, Pfizer) and sorafenib (Nexavar^®^, Bayer), can cross the cell membrane and inhibit this autocrine survival loop, however this loop is not accessed or inhibited by anti-VEGF antibodies [40]. Clinical data further supports the importance of the internal autocrine VEGF loop in HSC survival, as both sunitinib [42,43] and sorafenib [44] are myelosuppressive as monotherapies, whereas bevacizumab is not. Interestingly, mice treated *in vivo* with VEGF-Trap or an antibody directed against VEGFR2 experience significantly delayed hematopoietic recovery following treatment with sublethal irradiation [45,46]. This lag in hematopoietic reconstitution is not due to direct effects on HSC survival but rather is attributed to delayed regeneration of sinusoidal endothelial cells in the bone marrow, which is required for HSC self-renewal, survival, and differentiation [45,46]. 

VEGF secretion effectively mobilizes mononuclear myeloid cell from the bone marrow. These cells home to target organs, position themselves in perivascular regions, and promote angiogenesis through secretion of angiogenic cytokines [47]. Recent work also specifies an important function for HSCs in tumor metastasis. Circulating HSCs home to sites of future metastasis, where they secrete proteases and growth factors, effectively preparing the “soil” for the metastasizing tumor cells. Homing to the pre-metastatic niche is purported to require VEGFR1 signaling, as treatment with anti-VEGFR1 antibodies can prevent HSC homing and subsequent metastasis [48]. In summary, VEGF signaling is important in the maintenance and survival of HSCs and promotes their mobilization from the bone marrow and homing to target organs (Table 1).

PTN is also expressed in the adult bone marrow where it stimulates osteogenic differentiation at low concentrations [49]. PTN is crucial for the differentiation of many stem cell types including human embryonic stem cells [50], neural stem cells, late retinal progenitor cells [51], and myoblasts [52]. PTN also promotes nitric oxide (NO)-dependent mobilization and migration of endothelial progenitor cells [53]. These studies suggest that PTN functions primarily as a stem cell differentiation factor, but it is unknown what specific effects PTN has on HSC survival and differentiation.

## 3. Macrophages

Inflammatory cells comprise a major portion of the overall tumor mass and of these, macrophages represent an abundant and important cell type [54]. In fact, macrophages regulate the angiogenic switch in MMTV-PyMT tumors [55]. There are two basic classes of macrophages found in tumors. Classically activated (M1) macrophages are potent effector cells that produce pro-inflammatory cytokines (IL-6, IL-12, TNFα) and are capable of killing tumor cells. In contrast, alternatively activated (M2) macrophages secrete angiogenic and anti-inflammatory cytokines (IL-10, TGFβ, and VEGF) and therefore suppress the immune system and promote tumor progression (reviewed in [56]). The majority of macrophages in the tumor microenvironment display an M2 phenotype, as macrophage depletion inhibits tumor growth and metastasis in many pre-clinical models [55,57]. Interestingly, expression of VEGF restores tumor growth in macrophage-depleted animals, indicating that macrophage-derived VEGF is critical for tumor progression, angiogenesis, and invasion [58]. In addition, increased macrophage infiltration confers a poor prognosis in breast cancer, whereby an increase in macrophage hotspots correlates with decreased relapse-free and overall survival [59,60]. Recent studies also suggest that a distinct macrophage population assists in metastasis by facilitating tumor cell intravasation and extravasation [61,62]. Furthermore, depletion of macrophages after tumor cell seeding at the metastatic site is sufficient to significantly reduce metastatic burden in pre-clinical models of breast cancer [61]. This distinct macrophage population can be described as F480^+^CD11b^+^Gr1^-^CCR2^hi^CX3CR1^hi^ and VEGFR1^hi^, however, the VEGFR2 status of these macrophages has not been established.

VEGF stimulates macrophage chemotaxis [7] into the tumor microenvironment, and we and others have shown that anti-VEGF therapy can reduce macrophage infiltration in pre-clinical tumor models (Table 1) [63,64,65,66,67]. We found that macrophages harvested from a tumor-bearing animal express both VEGFR1 and VEGFR2, whereas those harvested from non-tumor bearing mice are VEGFR1^+^ but deficient in VEGFR2. Furthermore, when VEGFR2 is expressed, it becomes the dominant receptor driving VEGF-induced chemotaxis and specific blockade of VEGF:VEGFR2 interaction is sufficient to inhibit chemotaxis [63,64]. Interestingly, analysis of human peripheral blood from cancer patients and healthy volunteers revealed a population of VEGFR2^+^/CD45^bright^/CD14^+^ monocytes; this population was significantly more prominent in blood from cancer patients compared to that of healthy volunteers, confirming our pre-clinical findings [68]

**Table 1:** Summary of the effects of VEGF, pleiotrophin, and pre-clinical and clinical anti-VEGF therapy on the immune profile.

Cell Type	VEGF Effects	Pre-Clinical Anti-VEGF	Clinical Anti-VEGF	Pleiotrophin Effects
Hematopoietic stem cells (HSCs)	Regulates pluripotency, survival, and mobilization from the bone marrow [25,26]	Anti-VEGFR2 inhibits reconstitution following sublethal irradiation [30,31] Anti-VEGFR1 prevents mobilization and recruitment to pre-metastatic niche in a Lewis lung carcinoma model [33]	Sunitinib and sorafenib result in myelosuppression as monotherapies [27,28,29]	Unknown
Macrophages	Macrophage chemotaxis [7]	Reduces macrophage infiltration in multiple breast cancer and other cancer models [48,49,50,51,52]	Unknown	Induces macrophage VEGFR2 expression and promotes an angiogenic phenotype [48,54,55,56]
Myeloid derived suppressor cells (MDSCs)	Promotes differentiation into neutrophils, macrophages, and dendritic cells [59]	VEGFR2 specific inhibition decreases MDSC in MDA-MB-231 xenograft and MMTV-PyMT transgenic models [50] Inhibition of both VEGFR1 and VEGFR2 increases MDSC number in the tumor in MDA-MB-231 xenograft and MMTV-PyMT transgenic models [50]	Sunitinib decreases MDSCs in in patients with renal cell carcinoma (RCC) [91] Bevacizumab decreases MDSCs in patients with a variety of cancers [77]	Unknown
Neutrophils	Neutrophil chemotaxis [65]	VEGFR2 specific inhibition increases neutrophil infiltration into tumors in multiple breast cancer models [49,50] Inhibition of both VEGFR1 and VEGFR2 decrease neutrophil infiltration into tumors in multiple breast cancer models [49,50]	Unknown	Neutrophil chemotaxis [68]
Dendritic cells (DCs)	VEGF:VEGFR1 activation inhibits the differentiation of HSCs down the DC lineage [71,72] VEGF:VEGFR2 activation inhibits DC antigen presenting cell functions [73,74]	Specific inhibition of VEGF: VEGFR2 activation increases the number of mature dendritic cells in the MDA-MB-231 xenograft and 4T1 syngeneic breast cancer models [49,50]	Sorafenib reverses defects in DC maturation in patients with RCC [78] Bevacizumab reverses defects in DC maturation in colorectal cancer patients [78]	Unknown
Regulatory T-cells (Tregs)	VEGF expression can be correlated to high FoxP3 expression in breast carcinoma [83]	VEGFR2 specific inhibition decreases Tregs MMTV-PyMT transgenic model [50] Inhibition of both VEGFR1 and VEGFR2 increases Tregs in the MMTV-PyMT transgenic model [50]	Sunitinib decreased Tregs in patients with RCC [91]	Unknown

Specific inhibitors of the VEGF:VEGFR2 interaction significantly increase PTN expression in three separate pre-clinical tumor models, suggesting that VEGFR1 signaling may be important in this phenomenon [26]. Once expressed, PTN induces the expression of VEGFR2 on macrophages and can promote the transdifferentiation of macrophages into functional endothelial cells *in vitro* [63,69,70]. Furthermore, PTN may promote an angiogenic macrophage (M2) phenotype *in vitro* [71]. This suggests that while anti-VEGF therapy reduces macrophage infiltration into the tumor, compensating expression of PTN can promote an M2 phenotype in those macrophages that are either (1) in the tumor prior to therapy or (2) migrate into the tumor in response to a non-VEGF cytokine.

Interestingly, conditioned media from the ovarian cancer cell line SKOV3 is unable to induce the differentiation of monocytes into CD14^+^/VEGFR2^+^ cells, until these cells are forced to undergo EMT [71]. Conditioned media from SKOV3 cells with a mesenchymal phenotype contains increased levels of PTN and induces the differentiation of monocytes into CD14^+^/VEGFR2^+^ cells [71]. These CD14^+^/VEGFR2^+^ angiogenic monocytes are able to increase *in vitro* tube formation when co-cultured with endothelial cells and induce endothelial cell migration, indicating that VEGFR2^+^ monocytes may represent a sub-type of M2 macrophages [71]. Furthermore, addition of an anti-PTN blocking antibody to mesenchymal SKOV3 CM prevented the differentiation of primary monocytes into CD14^+^/VEGFR2^+^ angiogenic monocytes [71].

## 4. Myeloid Derived Suppressor Cells

Myeloid derived suppressor cells (MDSCs) are an immature and heterogeneous population of cells described by the expression CD11b^+^Gr1^+^CD14^–^. MDSCs expand during tumorigenesis, inflammation, and infection and suppress the adaptive immune system through inhibition of T-cell function. MDSC induced T-cell anomalies in tumors include antigen-specific T-cell tolerance, nonspecific suppression of T-cell function, and induction of T-cell apoptosis [72]. MDSCs exert all of these effects through the secretion of arginase, reactive oxygen, and reactive nitrogen species. MDSCs also secrete large amounts of MMP-9, which regulates the bioavailability of VEGF in the tumor microenvironment, thus MDSCs increase VEGF bioavailability and indirectly increase tumor angiogenesis [73]. Furthermore, VEGF increases the number of MDSCs by preventing the differentiation of these cells into neutrophils, macrophages, and dendritic cells [74]. 

CD11b^+^Gr1^+^ cells are able to mediate refractoriness to anti-VEGF therapy through secretion of the alternative angiogenic factor Bv8. Bv8 binds to and signals through two G-protein coupled receptors termed EG-VEGFR/PKR-1 and EG-VEGFR/PKR-2. Strikingly, refractoriness to anti-VEGF therapy is alleviated when mice are treated with a combination of anti-VEGF and anti-Bv8 therapy [75,76]. Following these studies, the effects of acute and long term anti-VEGF therapy on MDSC number and function has become a topic of significant interest. We examined these effects using different of anti-VEGF strategies and three separate pre-clinical mouse models [65]. Our results indicate the effects of anti-VEGF therapy on MDSC infiltration are model dependent (Table 1). In MDA-MB-231 xenografts and MMTV-PyMT transgenic tumors simultaneous blockade of both VEGFR1 and VEGFR2 increased MDSC infiltration, however, this did not hold true in 4T1 syngenic tumors, where all anti-VEGF strategies reduced MDSC accumulation [65]. A potential explanation of this phenomenon is the level of intra-tumoral cytokine levels. IL-1β levels are changed following anti-VEGF therapy and regulate MDSC infiltration in a bimodal manner. We found low levels of IL-1β (<5 pg/mg/protein) correlate with increased MDSC infiltration in the MDA-MB-231 and MMTV-PyMT models while elevated levels of IL-1β (>50 pg/mg/protein) following anti-VEGF therapy in the 4T1 model correlated with reduced MDSC infiltration [65]. 

## 5. Neutrophils

While many studies have focused on the function of tumor-associated macrophages in tumor progression, the function of tumor associated neutrophils has not been studied in detail. However, recent evidence indicates that neutrophils also participate actively in tumorigenesis through a number of mechanisms including the secretion of angiogenic growth factors (*i.e.*, VEGF) and matrix-metalloproteinases, such as MMP-9 [77,78,79]. In fact, neutrophil derived MMP-9 mediates the angiogenic switch in a mouse model of pancreatic cancer [77,78]. Neutrophils respond to chemotactic agents including CXCL8 (IL-8), G-CSF, and VEGF. Neutrophil migration toward VEGF is mediated by VEGFR1 activation [80]. Anti-VEGF therapy modulates neutrophil infiltration in an agent dependent manner, whereby agents that specifically block activation of VEGFR2 increase neutrophil infiltration. In contrast those that block activation of both VEGFR1 and VEGFR2 decrease neutrophil infiltration, indicating that VEGFR1 is the primary receptor involved in VEGF-induced neutrophil chemotaxis into tumors [64,65]. It is important to note, that the differentiation of MDSCs into neutrophils following VEGFR2-specific therapy may also contribute to neutrophil accumulation in this model. Alternatively, recent evidence indicates that macrophages can reduce neutrophil infiltration by inhibiting CXCL8-dependent chemotaxis [81]. Therefore, macrophage numbers often correlate inversely with neutrophil levels. We have found that in general anti-VEGF strategies reduce macrophage infiltration; however only selective inhibition of VEGF-activation of VEGFR2 by receptor specific agents resulted in marked accumulation of neutrophils [65,81]. Interestingly, neutrophil numbers in the blood are an independent prognostic indicator of response to anti-VEGF therapy in patients with renal cell carcinoma, whereby increased neutrophil number correlates with a decreased overall survival following anti-VEGF therapy [82].

There are very few studies examining the effects of PTN on neutrophil infiltration and function. However, there is some evidence that PTN can function as a neutrophil chemotactic agent [83]. 

## 6. Dendritic Cells

Dendritic cells (DC) are the most potent antigen presenting cells and are crucial for induction and maintenance of anti-tumor responses. Defects in antigen presentation by DCs related to abnormal differentiation and activation are regulated by many soluble factors in the tumor microenvironment [84]. VEGF was the first described tumor-derived factor shown to inhibit DC differentiation [85]. During physiologic DC differentiation, variations in VEGFR expression regulate maturation of DC from bone marrow progenitor cells to immature DC in peripheral blood and to mature antigen presenting DCs in secondary lymphoid organs [74,86,87]. VEGF activation of VEGFR1 on CD34^+^ early progenitor cells blocks activation of NF-κB, resulting in inhibition of HSC differentiation along the DC lineage [74,87]. However, inhibition of DC function, via impaired antigen presentation and blunted T-cell stimulation and proliferation result from VEGFR2 activation [86,88]. 

The effects of VEGF and VEGF-targeted therapies on tumors extend beyond the effects on angiogenesis. Previously, using MDA-MB-231 xenografts, we found an increase in mature DC in animals treated with r84, an anti-VEGF antibody that selectively inhibits VEGF binding to VEGFR2, but not bevacizumab, an antibody that inhibits VEGF from binding VEGFR1 and VEGFR2 [64]. Using the 4T1 model in immunocompetent animals, we found a similar effect after only one week of therapy, where inhibition of VEGF binding to VEGFR2 with mouse chimeric r84 reduced the number of total DC, but increased the mature fraction of these cells [64]. 

Clinical data support that VEGF can induce DC defects in cancer patients. First, in cancer patients, elevated systemic VEGF levels correlate with low DC frequencies [89]. Furthermore, patients with metastatic cancer have an increased proportion of immature DC, a population of cells not capable of stimulating T-cell responses [90]. Additionally, in certain types of cancer, DC differentiation has been shown to be negatively affected by VEGF, as demonstrated in pre-clinical models [91]. Despite this wealth of pre-clinical data the majority of anti-VEGF agents have minimal effect on DC activity in human patients [91,92,93]. However, this is not true in all cases. Select anti-VEGF agents (renal cell cancer/sorafenib and colorectal cancer/bevacizumab) can reverse deficits in DC maturation [90]. These data highlight the importance of VEGF in DC maturation in cancer patients and support the further investigation of the anti-VEGF therapy on DC subsets.

## 7. Regulatory T-cells

**Figure 1:**
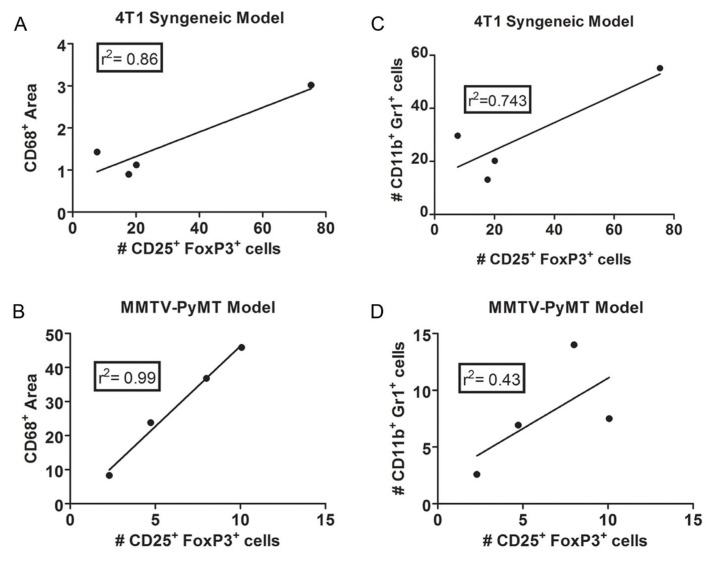
Macrophage infiltration correlates with the accumulation of CD25^+^ FoxP3^+^ regulatory T-cells in two pre-clinical models of breast cancer following anti-VEGF therapy. Mice in each experiment received therapy with either control antibody (C44), mouse-chimeric r84 (inhibits the VEGF: VEGFR2 interaction), sunitinib (inhibits VEGFR1, VEGFR2 PDGFRβ, c-kit), or GU81 (inhibits VEGFR1 and VEGFR2). (A, B) By linear regression analysis, changes intra-tumoral T_reg_ number following anti-VEGF therapy positively correlate with CD68^+^ macrophage levels after four weeks of therapy in the 4T1 syngeneic (A) and MMTV-PyMT transgenic breast cancer models (B). Each dot represents the mean for CD68^+^ area and the number of T_regs_ in each treatment group. (C, D) By linear regression analysis, changes in intra-tumor T_reg_ number positively correlate with the number of CD11b+ Gr1+ MDSCs following four weeks of therapy in the 4T1 syngenic (C) but not the MMTV-PyMT transgenic (D) breast cancer model. Each dot represents the mean for the number of MDSCs and T_regs_ in each treatment group.

Regulatory T-cells (T_reg_) are a specialized immunosuppressive population of T-cells defined by the expression of CD25 and FoxP3. T_regs_ are produced by the immune system to control self-tolerance and prevent autoimmunity and exert these effects through the secretion of anti-inflammatory cytokines such as TGFβ and IL-10 [94]. Tumors take advantage of this regulatory mechanism to prevent anti-tumor responses. They recruit and expand naturally occurring T_reg_ populations and induce the formation of new T_reg_ cohorts, which specifically recognize tumor-associated antigens. In fact, high FoxP3 expression is associated with increased TGFβ and VEGF levels, invasiveness, and metastasis in infiltrating breast carcinoma, indicating that FoxP3 expression can be used as a prognostic indicator [95]. TGFβ is reported to increase VEGF expression, explaining the correlation in these two parameters [96]. We have examined the effects of anti-VEGF therapy on T_reg_ accumulation in two immunocompetent mouse models and have found that the effects are model dependent. In MMTV-PyMT tumors, anti-VEGF therapy that specifically inhibits VEGFR2 signaling decreases T_reg_ accumulation, whereas blockade of both VEGFR1 and VEGFR2 signaling actually increases T_reg_ infiltration. In contrast, all anti-VEGF therapies inhibit T_reg_ accumulation in 4T1 tumors [65]. Recent evidence indicates that T_reg_ infiltration may be mediated by myeloid cells in the tumor microenvironment, whereby myeloid cell depletion can reduce T_reg_ homing to tumors [97]. Furthermore, macrophages are the key cell type responsible for T_reg_ expansion in certain immunosuppressive settings [98]. Re-examination and analysis of our previous data revealed that macrophage infiltration correlates with T_reg_ accumulation in the PyMT-MMTV (r^2^ = 0.99) and 4T1 (r^2^ = 0.86) models following anti-VEGF therapy (Figure 1A, B). Furthermore, MDSC number correlates with T_reg_ accumulation in 4T1 (r^2^ = 0.743) but not MMTV-PyMT tumors (r^2^ = 0.43) (Figure 1C, D). Examination of multiple cytokines revealed that only IFN-γ correlated consistently with T_reg_ number [65]. 

There are no studies which directly examine the effects of PTN on Treg cells; however, there are numerous studies, which suggest that PTN has a profound influence on macrophage phenotype. Therefore, PTN may indirectly affect Tregs in the tumor microenvironment through modulation of macrophage infiltration and phenotype.

## 8. Anti-VEGF Therapy in Breast Cancer

Bevacizumab, a humanized monoclonal antibody that binds human VEGF and prevents VEGF from binding VEGFR1 and VEGFR2, was approved for the treatment of metastatic HER2/NEU-negative breast cancer in 2008 [99]. The most relevant evidence for the efficacy of bevacizumab in the treatment of metastatic breast cancer comes from two large phase III clinical trials. In the first study, patients treated with the combination of capecitabine plus bevacizumab demonstrated increased overall response rate compared to capecitabine alone [100]. The next major phase III clinical trial prior to the FDA approval of bevacizumab was the ECOG 2100 trial [101]. Patients treated with paclitaxel plus bevacizumab had prolonged progression-free survival as compared to those treated with paclitaxel alone (11.8 *versus* 5.9 months). However, there were no differences in overall survival between the two groups. Sub-group analysis revealed that patients with the *VEGF-2578 AA* genotype or *VEGF-1154 A* allele had prolonged overall survival in the combination arm compared with the alternate genotypes. Additionally, expression of the genotypes *VEGF-634 CC* and *VEGF-1498 TT* were associated with significantly less grade 3 or 4 hypertension compared with the alternate genotypes [102], demonstrating the need for molecular characterization of tumors to maximize therapeutic benefit of anti-VEGF therapy. At this time, few studies have evaluated the effects of VEGF and anti-VEGF therapy on immune cells in cancer. In patients with metastatic renal cell carcinoma, treatment with sunitinib, a receptor tyrosine kinase inhibitor of VEGFR1, VEGFR2, platelet-derived growth factor, c-Kit, Flt3 and CSF-1, resulted in significant reduction in MDSCs and correlated with reversal of type 1 T-cell suppression [103]. Furthermore, MDSC reduction correlated with a reduction of CD3^+^CD4^+^CD25^hi^FoxP3^+^ T_reg_ cells. However, no correlation existed between change in tumor burden and change in T_reg_ or MDSC infiltration, suggesting an alteration in the phenotype of these cells. A recent study from Duke evaluated the effect of bevacizumab therapy on immune cells in patients with a variety of cancers, including breast cancer [90]. Compared to healthy volunteers, cancer patients had deficits in DC maturation and an accumulation of immature myeloid cells. Interestingly, the number of immature myeloid cells correlated with serum VEGF levels. Furthermore, bevacizumab treatment reduced the number of immature myeloid cells in the blood and enhanced *in vitro* DC immune function in cancer patients [90]. These data suggest a potential to modulate antitumor immunity by reversing tumor-induced immunosuppression with anti-VEGF therapy [103]. An increased understanding of the effects of anti-VEGF therapy on immune cells is critically important for patients with breast cancer, as many of these cell types have been implicated as contributors to resistance. , Furthermore, preliminary clinical and pre-clinical data suggests that patients could be accessed for response early during the course of anti-VEGF therapy by evaluating changes in immune cell numbers and phenotype in the blood.

## 9. Conclusions

It has long been recognized that VEGF is critical for tumor progression, where it serves as an important angiogenic factor in the tumor microenvironment [104]. This information along with the observation that tumors cannot grow beyond the size of 1–2 mm^3^ in the absence of angiogenesis [4] has led to the development of a number of anti-cancer agents that inhibit the VEGF pathway [5]. However, it has become clear that VEGFRs are expressed on not only endothelial and tumor cells but also various immune cells in the tumor microenvironment, and therefore it is important to understand the effects of anti-VEGF therapy on all cells types. 

Pre-clinical data suggests that treatment with inhibitors such as r84, which specifically inhibits VEGF signaling through VEGFR2, results in an improved immune profile compared to broader spectrum inhibitors [64,65]. Treatment with r84 decreased the number of immunosuppressive MDSC and T_regs_ and increased the number of mature DC in three separate pre-clinical models. Furthermore, this improvement in immune profile was not reproducible with any of the other anti-VEGF agents tested, including sunitinib and bevacizumab [64,65]. In fact, treatment with bevacizumab actually increased the number of tumor-associated MDSCs in the MDA-MB-231 xenograft model [65]. 

Finally it is important to note that not all tumors are sensitive or will remain sensitive to anti-VEGF therapy [10]. We must, therefore, investigate alternative angiogenic and immunosuppressive pathways, such as PTN signaling pathways that may promote resistance to anti-VEGF therapy. We have preliminary evidence that anti-VEGF therapy can influence PTN expression and signaling and anticipate that combination of anti-VEGF with anti-PTN therapy will dramatically augment anti-tumor effects by reducing angiogenesis and improving the immunogenicity of tumor cells. 

## References

[1] American Cancer Society (2009). Cancer Facts and Figures 2009.

[2] Tysnes B.B., Bjerkvig R. (2007). Cancer initiation and progression: involvement of stem cells and the microenvironment. Biochim. Biophys. Acta.

[3] DeNardo D.G., Coussens L.M. (2007). Inflammation and breast cancer. Balancing immune response: crosstalk between adaptive and innate immune cells during breast cancer progression. Breast Cancer Res..

[4] Folkman J. (1971). Tumor angiogenesis: therapeutic implications. N. Engl. J. Med..

[5] Dvorak H.F. (2002). Vascular permeability factor/vascular endothelial growth factor: a critical cytokine in tumor angiogenesis and a potential target for diagnosis and therapy. J. Clin. Oncol..

[6] Pradeep C.R., Sunila E.S., Kuttan G. (2005). Expression of vascular endothelial growth factor (VEGF) and VEGF receptors in tumor angiogenesis and malignancies. Integr. Cancer Ther..

[7] Sawano A., Iwai S., Sakurai Y., Ito M., Shitara K., Nakahata T., Shibuya M. (2001). Flt-1, vascular endothelial growth factor receptor 1, is a novel cell surface marker for the lineage of monocyte–macrophages in humans. Blood.

[8] Lee T.H., Seng S., Sekine M., Hinton C., Fu Y., Avraham H.K., Avraham S. (2007). Vascular endothelial growth factor mediates intracrine survival in human breast carcinoma cells through internally expressed VEGFR1/FLT1. PLoS Med..

[9] Yang A.D., Camp E.R., Fan F., Shen L., Gray M.J., Liu W., Somcio R., Bauer T.W., Wu Y., Hicklin D.J., Ellis L.M. (2006). Vascular endothelial growth factor receptor-1 activation mediates epithelial to mesenchymal transition in human pancreatic carcinoma cells. Cancer Res..

[10] Ellis L.M., Hicklin D.J. (2008). Pathways mediating resistance to vascular endothelial growth factor-targeted therapy. Clin. Cancer Res..

[11] Grepin R., Pages G. (2010). Molecular mechanisms of resistance to tumour anti-angiogenic strategies. J. Oncol..

[12] Berardi A.C., Wang A., Abraham J., Scadden D.T. (1995). Basic fibroblast growth factor mediates its effects on committed myeloid progenitors by direct action and has no effect on hematopoietic stem cells. Blood.

[13] Byrd V.M., Ballard D.W., Miller G.G., Thomas J.W. (1999). Fibroblast growth factor-1 (FGF-1) enhances IL-2 production and nuclear translocation of NF-kappaB in FGF receptor-bearing Jurkat T cells. J. Immunol..

[14] Kitayama J., Nagawa H., Yasuhara H., Tsuno N., Kimura W., Shibata Y., Muto T. (1994). Suppressive effect of basic fibroblast growth factor on transendothelial emigration of CD4(+) T-lymphocyte. Cancer Res..

[15] Nakayama T., Mutsuga N., Tosato G. (2007). Effect of fibroblast growth factor 2 on stromal cell-derived factor 1 production by bone marrow stromal cells and hematopoiesis. J. Natl. Cancer Inst..

[16] Ribatti D., Nico B., Vacca A., Roncali L., Presta M. (1999). Endogenous and exogenous fibroblast growth factor-2 modulate wound healing in the chick embryo chorioallantoic membrane. Angiogenesis.

[17] Takagi S., Takahashi K., Katsura Y., Matsuoka T., Ohsaka A. (2000). Basic fibroblast growth factor modulates the surface expression of effector cell molecules and primes respiratory burst activity in human neutrophils. Acta Haematol..

[18] Zhao X.M., Citrin B.S., Miller G.G., Frist W.H., Merrill W.H., Fischell T.A., Atkinson J.B., Yeoh T.K. (1995). Association of acidic fibroblast growth factor and untreated low grade rejection with cardiac allograft vasculopathy. Transplantation.

[19] Lewis C.E., De Palma M., Naldini L. (2007). Tie2-expressing monocytes and tumor angiogenesis: regulation by hypoxia and angiopoietin-2. Cancer Res..

[20] Lewis C.E., Hughes R. (2007). Inflammation and breast cancer. Microenvironmental factors regulating macrophage function in breast tumours: hypoxia and angiopoietin-2. Breast Cancer Res..

[21] Lin Y.L., Liang Y.C., Chiang B.L. (2007). Placental growth factor down-regulates type 1 T helper immune response by modulating the function of dendritic cells. J. Leukoc. Biol..

[22] Selvaraj S.K., Giri R.K., Perelman N., Johnson C., Malik P., Kalra V.K. (2003). Mechanism of monocyte activation and expression of proinflammatory cytochemokines by placenta growth factor. Blood.

[23] Burger J.A., Kipps T.J. (2006). CXCR4: a key receptor in the crosstalk between tumor cells and their microenvironment. Blood.

[24] Schulz C., von Andrian U.H., Massberg S. (2009). Hematopoietic stem and progenitor cells: their mobilization and homing to bone marrow and peripheral tissue. Immunol. Res..

[25] Perez-Pinera P., Chang Y., Deuel T.F. (2007). Pleiotrophin, a multifunctional tumor promoter through induction of tumor angiogenesis, remodeling of the tumor microenvironment, and activation of stromal fibroblas. Cell Cycle.

[26] Lynn K.D., Roland C.L., Brekken R.A. (2010).

[27] Rauvala H. (1989). An 18-kd heparin-binding protein of developing brain that is distinct from fibroblast growth factors. EMBO J..

[28] Silos-Santiago I., Yeh H.J., Gurrieri M.A., Guillerman R.P., Li Y.S., Wolf J., Snider W., Deuel T.F. (1996). Localization of pleiotrophin and its mRNA in subpopulations of neurons and their corresponding axonal tracts suggests important roles in neural-glial interactions during development and in maturity. J. Neurobiol..

[29] Vanderwinden J.M., Mailleux P., Schiffmann S.N., Vanderhaeghen J.J. (1992). Cellular distribution of the new growth factor pleiotrophin (HB-GAM) mRNA in developing and adult rat tissues. Anat. Embryol. (Berl.).

[30] Yeh H.J., He Y.Y., Xu J., Hsu C.Y., Deuel T.F. (1998). Upregulation of pleiotrophin gene expression in developing microvasculature, macrophages, and astrocytes after acute ischemic brain injury. J. Neurosci..

[31] Courty J., Dauchel M.C., Caruelle D., Perderiset M., Barritault D. (1991). Mitogenic properties of a new endothelial cell growth factor related to pleiotrophin. Biochem. Biophys. Res. Commun..

[32] Fang W., Hartmann N., Chow D.T., Riegel A.T., Wellstein A. (1992). Pleiotrophin stimulates fibroblasts and endothelial and epithelial cells and is expressed in human cancer. J. Biol. Chem..

[33] Laaroubi K., Delbe J., Vacherot F., Desgranges P., Tardieu M., Jaye M., Barritault D., Courty J. (1994). Mitogenic and *in vitro* angiogenic activity of human recombinant heparin affin regulatory peptide. Growth Factors.

[34] Milner P.G., Li Y.S., Hoffman R.M., Kodner C.M., Siegel N.R., Deuel T.F. (1989). A novel 17 kD heparin-binding growth factor (HBGF-8) in bovine uterus: purification and N-terminal amino acid sequence. Biochem. Biophys. Res. Commun..

[35] Perez-Pinera P., Alcantara S., Dimitrov T., Vega J.A., Deuel T.F. (2006). Pleiotrophin disrupts calcium-dependent homophilic cell-cell adhesion and initiates an epithelial-mesenchymal transition. Proc. Natl. Acad. Sci. USA.

[36] Riegel A.T., Wellstein A. (1994). The potential role of the heparin-binding growth factor pleiotrophin in breast cancer. Breast Cancer Res. Treat..

[37] Choudhuri R., Zhang H.T., Donnini S., Ziche M., Bicknell R. (1997). An angiogenic role for the neurokines midkine and pleiotrophin in tumorigenesis. Cancer Res..

[38] Zhang N., Zhong R., Wang Z.Y., Deuel T.F. (1997). Human breast cancer growth inhibited *in vivo* by a dominant negative pleiotrophin mutant. J. Biol. Chem..

[39] Chang Y., Zuka M., Perez-Pinera P., Astudillo A., Mortimer J., Berenson J.R., Deuel T.F. (2007). Secretion of pleiotrophin stimulates breast cancer progression through remodeling of the tumor microenvironment. Proc. Natl. Acad. Sci. USA.

[40] Gerber H.P., Malik A.K., Solar G.P., Sherman D., Liang X.H., Meng G., Hong K., Marsters J.C., Ferrara N. (2002). VEGF regulates haematopoietic stem cell survival by an internal autocrine loop mechanism. Nature.

[41] Robertson S., Kennedy M., Keller G. (1999). Hematopoietic commitment during embryogenesis. Ann. N. Y. Acad. Sci..

[42] Demetri G.D., van Oosterom A.T., Garrett C.R., Blackstein M.E., Shah M.H., Verweij J., McArthur G., Judson I.R., Heinrich M.C., Morgan J.A., Desai J., Fletcher C.D., George S., Bello C.L., Huang X., Baum C.M., Casali P.G. (2006). Efficacy and safety of sunitinib in patients with advanced gastrointestinal stromal tumour after failure of imatinib: a randomised controlled trial. Lancet.

[43] Motzer R.J., Michaelson M.D., Rosenberg J., Bukowski R.M., Curti B.D., George D.J., Hudes G.R., Redman B.G., Margolin K.A., Wilding G. (2007). Sunitinib efficacy against advanced renal cell carcinoma. J. Urol..

[44] (2009). N*exavar (sorafenib) package insert*, 17.5 FDA-approved patient labeling.

[45] Hooper A.T., Butler J.M., Nolan D.J., Kranz A., Iida K., Kobayashi M., Kopp H.G., Shido K., Petit I., Yanger K., James D., Witte L., Zhu Z., Wu Y., Pytowski B., Rosenwaks Z., Mittal V., Sato T.N., Rafii S. (2009). Engraftment and reconstitution of hematopoiesis is dependent on VEGFR2-mediated regeneration of sinusoidal endothelial cells. Cell Stem Cell.

[46] Kopp H.G., Hooper A.T., Avecilla S.T., Rafii S. (2009). Functional heterogeneity of the bone marrow vascular niche. Ann. N. Y. Acad. Sci..

[47] Grunewald M., Avraham I., Dor Y., Bachar-Lustig E., Itin A., Jung S., Chimenti S., Landsman L., Abramovitch R., Keshet E. (2006). VEGF-induced adult neovascularization: recruitment, retention, and role of accessory cells. Cell.

[48] Kaplan R.N., Riba R.D., Zacharoulis S., Bramley A.H., Vincent L., Costa C., MacDonald D.D., Jin D.K., Shido K., Kerns S.A., Zhu Z., Hicklin D., Wu Y., Port J.L., Altorki N., Port E.R., Ruggero D., Shmelkov S.V., Jensen K.K., Rafii S., Lyden D. (2005). VEGFR1-positive haematopoietic bone marrow progenitors initiate the pre-metastatic niche. Nature.

[49] Tare R.S., Oreffo R.O., Clarke N.M., Roach H.I. (2002). Pleiotrophin/Osteoblast-stimulating factor 1: dissecting its diverse functions in bone formation. J. Bone Miner. Res..

[50] Vazin T., Becker K.G., Chen J., Spivak C.E., Lupica C.R., Zhang Y., Worden L., Freed W.J. (2009). A novel combination of factors, termed SPIE, which promotes dopaminergic neuron differentiation from human embryonic stem cells. PLoS One.

[51] Roger J., Brajeul V., Thomasseau S., Hienola A., Sahel J.A., Guillonneau X., Goureau O. (2006). Involvement of Pleiotrophin in CNTF-mediated differentiation of the late retinal progenitor cells. Dev. Biol..

[52] Caruelle D., Mazouzi Z., Husmann I., Delbe J., Duchesnay A., Gautron J., Martelly I., Courty J. (2004). Upregulation of HARP during *in vitro* myogenesis and rat soleus muscle regeneration. J. Muscle Res. Cell Motil..

[53] Heiss C., Wong M.L., Block V.I., Lao D., Real W.M., Yeghiazarians Y., Lee R.J., Springer M.L. (2008). Pleiotrophin induces nitric oxide dependent migration of endothelial progenitor cells. J. Cell. Physiol..

[54] Pollard J.W. (2004). Tumour-educated macrophages promote tumour progression and metastasis. Nat. Rev. Cancer..

[55] Lin E.Y., Li J.F., Gnatovskiy L., Deng Y., Zhu L., Grzesik D.A., Qian H., Xue X.N., Pollard J.W. (2006). Macrophages regulate the angiogenic switch in a mouse model of breast cancer. Cancer Res..

[56] Allavena P., Sica A., Garlanda C., Mantovani A. (2008). The Yin-Yang of tumor-associated macrophages in neoplastic progression and immune surveillance. Immunol. Rev..

[57] Zeisberger S.M., Odermatt B., Marty C., Zehnder-Fjallman A.H., Ballmer-Hofer K., Schwendener R.A. (2006). Clodronate-liposome-mediated depletion of tumour-associated macrophages: a new and highly effective antiangiogenic therapy approach. Br. J. Cancer.

[58] Lin E.Y., Li J.F., Bricard G., Wang W., Deng Y., Sellers R., Porcelli S.A., Pollard J.W. (2007). Vascular endothelial growth factor restores delayed tumor progression in tumors depleted of macrophages. Mol. Oncol..

[59] Leek R.D., Lewis C.E., Whitehouse R., Greenall M., Clarke J., Harris A.L. (1996). Association of macrophage infiltration with angiogenesis and prognosis in invasive breast carcinoma. Cancer Res..

[60] Leek R. D., Harris A. L. (2002). Tumor-associated macrophages in breast cancer. J. Mammary Gland Biol. Neoplasia.

[61] Qian B., Deng Y., Im J.H., Muschel R.J., Zou Y., Li J., Lang R.A., Pollard J.W. (2009). A distinct macrophage population mediates metastatic breast cancer cell extravasation, establishment and growth. PLoS One.

[62] Wyckoff J.B., Wang Y., Lin E.Y., Li J.F., Goswami S., Stanley E.R., Segall J.E., Pollard J.W., Condeelis J. (2007). Direct visualization of macrophage-assisted tumor cell intravasation in mammary tumors. Cancer Res..

[63] Dineen S.P., Lynn K.D., Holloway S.E., Miller A.F., Sullivan J.P., Shames D.S., Beck A.W., Barnett C.C., Fleming J.B., Brekken R.A. (2008). Vascular endothelial growth factor receptor 2 mediates macrophage infiltration into orthotopic pancreatic tumors in mice. Cancer Res..

[64] Roland C.L., Dineen S.P., Lynn K.D., Sullivan L.A., Dellinger M.T., Sadegh L., Sullivan J.P., Shames D.S., Brekken R.A. (2009). Inhibition of vascular endothelial growth factor reduces angiogenesis and modulates immune cell infiltration of orthotopic breast cancer xenografts. Mol. Cancer Ther..

[65] Roland C.L., Lynn K.D., Toombs J.E., Dineen S.P., Udugamasooriya D.G., Brekken R.A. (2009). Cytokine levels correlate with immune cell infiltration after anti-VEGF therapy in preclinical mouse models of breast cancer. PLoS One.

[66] Salnikov A.V., Heldin N.E., Stuhr L.B., Wiig H., Gerber H., Reed R.K., Rubin K. (2006). Inhibition of carcinoma cell-derived VEGF reduces inflammatory characteristics in xenograft carcinoma. Int. J. Cancer.

[67] Whitehurst B., Flister M.J., Bagaitkar J., Volk L., Bivens C.M., Pickett B., Castro-Rivera E., Brekken R.A., Gerard R.D., Ran S. (2007). Anti-VEGF-A therapy reduces lymphatic vessel density and expression of VEGFR-3 in an orthotopic breast tumor model. Int. J. Cancer.

[68] Vroling L., Yuana Y., Schuurhuis G.J., van Hinsbergh V.W., Gundy C., de Haas R., van Cruijsen H., Boven E., Hoekman K., Broxterman H.J. (2007). VEGFR2 expressing circulating (progenitor) cell populations in volunteers and cancer patients. Thromb. Haemost..

[69] Chen H., Campbell R.A., Chang Y., Li M., Wang C.S., Li J., Sanchez E., Share M., Steinberg J., Berenson A., Shalitin D., Zeng Z., Gui D., Perez-Pinera P., Berenson R.J., Said J., Bonavida B., Deuel T.F., Berenson J.R. (2009). Pleiotrophin produced by multiple myeloma induces transdifferentiation of monocytes into vascular endothelial cells: a novel mechanism of tumor-induced vasculogenesis. Blood.

[70] Sharifi B.G., Zeng Z., Wang L., Song L., Chen H., Qin M., Sierra-Honigmann M.R., Wachsmann-Hogiu S., Shah P.K. (2006). Pleiotrophin induces transdifferentiation of monocytes into functional endothelial cells. Arterioscler. Thromb. Vasc. Biol..

[71] Collino F., Revelli A., Massobrio M., Katsaros D., Schmitt-Ney M., Camussi G., Bussolati B. (2009). Epithelial-mesenchymal transition of ovarian tumor cells induces an angiogenic monocyte cell population. Exp. Cell Res..

[72] Nagaraj S., Gabrilovich D.I. (2008). Tumor escape mechanism governed by myeloid-derived suppressor cells. Cancer Res..

[73] Yang L., DeBusk L.M., Fukuda K., Fingleton B., Green-Jarvis B., Shyr Y., Matrisian L.M., Carbone D.P., Lin P.C. (2004). Expansion of myeloid immune suppressor Gr+CD11b+ cells in tumor-bearing host directly promotes tumor angiogenesis. Cancer Cell.

[74] Gabrilovich D., Ishida T., Oyama T., Ran S., Kravtsov V., Nadaf S., Carbone D.P. (1998). Vascular endothelial growth factor inhibits the development of dendritic cells and dramatically affects the differentiation of multiple hematopoietic lineages *in vivo*. Blood.

[75] Shojaei F., Wu X., Malik A.K., Zhong C., Baldwin M.E., Schanz S., Fuh G., Gerber H.P., Ferrara N. (2007). Tumor refractoriness to anti-VEGF treatment is mediated by CD11b+Gr1+ myeloid cells. Nat. Biotechnol..

[76] Shojaei F., Wu X., Zhong C., Yu L., Liang X.H., Yao J., Blanchard D., Bais C., Peale F.V., van Bruggen N., Ho C., Ross J., Tan M., Carano R.A., Meng Y.G., Ferrara N. (2007). Bv8 regulates myeloid-cell-dependent tumour angiogenesis. Nature.

[77] Bergers G., Brekken R., McMahon G., Vu T.H., Itoh T., Tamaki K., Tanzawa K., Thorpe P., Itohara S., Werb Z., Hanahan D. (2000). Matrix metalloproteinase-9 triggers the angiogenic switch during carcinogenesis. Nat. Cell Biol..

[78] Nozawa H., Chiu C., Hanahan D. (2006). Infiltrating neutrophils mediate the initial angiogenic switch in a mouse model of multistage carcinogenesis. Proc. Natl. Acad. Sci. USA.

[79] Scapini P., Morini M., Tecchio C., Minghelli S., Di Carlo E., Tanghetti E., Albini A., Lowell C., Berton G., Noonan D.M., Cassatella M.A. (2004). CXCL1/macrophage inflammatory protein-2-induced angiogenesis *in vivo* is mediated by neutrophil-derived vascular endothelial growth factor-A. J. Immunol..

[80] Ancelin M., Chollet-Martin S., Herve M.A., Legrand C., El Benna J., Perrot-Applanat M. (2004). Vascular endothelial growth factor VEGF189 induces human neutrophil chemotaxis in extravascular tissue via an autocrine amplification mechanism. Lab. Invest..

[81] Pahler J.C., Tazzyman S., Erez N., Chen Y.Y., Murdoch C., Nozawa H., Lewis C.E., Hanahan D. (2008). Plasticity in tumor-promoting inflammation: impairment of macrophage recruitment evokes a compensatory neutrophil response. Neoplasia.

[82] Heng D.Y., Xie W., Regan M.M., Warren M.A., Golshayan A.R., Sahi C., Eigl B.J., Ruether J.D., Cheng T., North S., Venner P., Knox J.J., Chi K.N., Kollmannsberger C., McDermott D.F., Oh W.K., Atkins M.B., Bukowski R.M., Rini B.I., Choueiri T.K. (2009). Prognostic factors for overall survival in patients with metastatic renal cell carcinoma treated with vascular endothelial growth factor-targeted agents: results from a large, multicenter study. J. Clin. Oncol..

[83] Ochiai K., Muramatsu H., Yamamoto S., Ando H., Muramatsu T. (2004). The role of midkine and pleiotrophin in liver regeneration. Liver Int..

[84] Gabrilovich D. (2004). Mechanisms and functional significance of tumour-induced dendritic-cell defects. Nat. Rev. Immunol..

[85] Gabrilovich D.I., Chen H.L., Girgis K.R., Cunningham H.T., Meny G.M., Nadaf S., Kavanaugh D., Carbone D.P. (1996). Production of vascular endothelial growth factor by human tumors inhibitis the functional maturation of dendritic cells. Nat. Med..

[86] Huang Y., Chen X., Dikov M.M., Novitskiy S.V., Mosse C.A., Yang L., Carbone D.P. (2007). Distinct roles of VEGFR-1 and VEGFR-2 in the aberrant hematopoiesis associated with elevated levels of VEGF. Blood.

[87] Oyama T., Ran S., Ishida T., Nadaf S., Kerr L., Carbone D. P., Gabrilovich D. I. (1998). Vascular endothelial growth factor affects dendritic cell maturation through the inhibition of nuclear factor-kappa B activation in hemopoietic progenitor cells. J. Immunol..

[88] Mimura K., Kono K., Takahashi A., Kawaguchi Y., Fujii H. (2007). Vascular endothelial growth factor inhibits the function of human mature dendritic cells mediated by VEGF receptor-2. Cancer Immunol. Immunother..

[89] Almand B., Resser J.R., Lindman B., Nadaf S., Clark J.I., Kwon E.D., Carbone D.P., Gabrilovich D.I. (2000). Clinical significance of defective dendritic cell differentiation in cancer. Clin. Cancer Res..

[90] Osada T., Chong G., Tansik R., Hong T., Spector N., Kumar R., Hurwitz H.I., Dev I., Nixon A.B., Lyerly H.K., Clay T., Morse M.A. (2008). The effect of anti-VEGF therapy on immature myeloid cell and dendritic cells in cancer patients. Cancer Immunol. Immunother..

[91] Fricke I., Mirza N., Dupont J., Lockhart C., Jackson A., Lee J.H., Sosman J.A., Gabrilovich D.I. (2007). Vascular endothelial growth factor-trap overcomes defects in dendritic cell differentiation but does not improve antigen-specific immune responses. Clin. Cancer Res..

[92] Hipp M.M., Hilf N., Walter S., Werth D., Brauer K.M., Radsak M.P., Weinschenk T., Singh-Jasuja H., Brossart P. (2008). Sorafenib, but not sunitinib, affects function of dendritic cells and induction of primary immune responses. Blood.

[93] van Cruijsen H., Hoekman K., Stam A.G., van den Eertwegh A.J., Kuenen B.C., Scheper R.J., Giaccone G., de Gruijl T.D. (2007). Defective differentiation of myeloid and plasmacytoid dendritic cells in advanced cancer patients is not normalized by tyrosine kinase inhibition of the vascular endothelial growth factor receptor. Clin. Dev. Immunol..

[94] Dieckmann D., Plottner H., Berchtold S., Berger T., Schuler G. (2001). Ex vivo isolation and characterization of CD4(+)CD25(+) T cells with regulatory properties from human blood. J. Exp. Med..

[95] Gupta S., Joshi K., Wig J.D., Arora S.K. (2007). Intratumoral FOXP3 expression in infiltrating breast carcinoma: Its association with clinicopathologic parameters and angiogenesis. Acta Oncol..

[96] Donovan D., Harmey J.H., Toomey D., Osborne D.H., Redmond H.P., Bouchier-Hayes D.J. (1997). TGF beta-1 regulation of VEGF production by breast cancer cells. Ann. Surg. Oncol..

[97] Qin F.X. (2009). Dynamic behavior and function of Foxp3+ regulatory T cells in tumor bearing host. Cell Mol. Immunol..

[98] Perruche S., Zhang P., Liu Y., Saas P., Bluestone J.A., Chen W. (2008). CD3-specific antibody-induced immune tolerance involves transforming growth factor-beta from phagocytes digesting apoptotic T cells. Nat. Med..

[99] Cortes-Funes H. (2009). The role of antiangiogenesis therapy: bevacizumab and beyond. Clin. Transl. Oncol..

[100] Miller K.D., Chap L.I., Holmes F.A., Cobleigh M.A., Marcom P.K., Fehrenbacher L., Dickler M., Overmoyer B.A., Reimann J.D., Sing A.P., Langmuir V., Rugo H.S. (2005). Randomized phase III trial of capecitabine compared with bevacizumab plus capecitabine in patients with previously treated metastatic breast cancer. J. Clin. Oncol..

[101] Miller K., Wang M., Gralow J., Dickler M., Cobleigh M., Perez E.A., Shenkier T., Cella D., Davidson N.E. (2007). Paclitaxel plus bevacizumab *versus* paclitaxel alone for metastatic breast cancer. N. Engl. J. Med..

[102] Schneider B.P., Wang M., Radovich M., Sledge G.W., Badve S., Thor A., Flockhart D.A., Hancock B., Davidson N., Gralow J., Dickler M., Perez E.A., Cobleigh M., Shenkier T., Edgerton S., Miller K.D. (2008). Association of vascular endothelial growth factor and vascular endothelial growth factor receptor-2 genetic polymorphisms with outcome in a trial of paclitaxel compared with paclitaxel plus bevacizumab in advanced breast cancer: ECOG 2100. J. Clin. Oncol..

[103] Ko J.S., Zea A.H., Rini B.I., Ireland J.L., Elson P., Cohen P., Golshayan A., Rayman P.A., Wood L., Garcia J., Dreicer R., Bukowski R., Finke J.H. (2009). Sunitinib mediates reversal of myeloid-derived suppressor cell accumulation in renal cell carcinoma patients. Clin. Cancer Res..

[104] Connolly D.T., Heuvelman D.M., Nelson R., Olander J.V., Eppley B.L., Delfino J.J., Siegel N.R., Leimgruber R.M., Feder J. (1989). Tumor vascular permeability factor stimulates endothelial cell growth and angiogenesis. J. Clin. Invest..

